# Snakebite envenomation in children: An ongoing burden in Morocco

**DOI:** 10.1016/j.amsu.2022.103574

**Published:** 2022-04-02

**Authors:** Meryem Essafti, Mohamed Fajri, Chadi Rahmani, Sihami Abdelaziz, Youssef Mouaffak, Said Younous

**Affiliations:** a Anesthesia and Critical Care Department, Pediatric Intensive Care Unit, CHU Mohamed VI, Marrakesh, Morocco; b Faculty of Medicine and Pharmacy of Marrakesh, Morocco

**Keywords:** Snake bites, Pediatric, Specific immunotherapy, Fasciotomy, Prevention

## Abstract

**Introduction:**

Snakebites are a leading cause of mortality and permanent disabilities especially among children in tropical countries and rural areas such as Morocco. Thus, a nationwide management protocol including specific antivenom therapy along with prevention strategies was implemented to reduce the overall snakebites morbimortality.

**Patients and methods:**

Our retrospective study aimed to describe the clinical aspects of snakebite envenomation before and after the implementation of this protocol in children admitted to the pediatric intensive care unit (PICU) in Marrakesh-Morocco for a period of 11 years.

**Results:**

A total of 75 cases were included and were mostly male (70%) with a mean age of 10 years old. Most envenomations were mild or severe (75%) and often occurred during outdoor activities in limb extremities. Altered hemostasis frequently occurred in 67% of cases but was rarely associated with severe exteriorized hemorrhage. Moderate anemia and PNN- predominant leukocytosis were often observed at admission (52.2% and 58%) but quickly tended to normalize before 48 h. Local symptoms were the main dread as they quickly evolve to a compartment syndrome and necrosis in the absence of antivenom therapy. Fasciotomy was performed in 33% of cases while 5 children required limb amputation. Antivenom administration (n = 39) was statistically significant for rapid improvement in hemostasis disorders, reduced blood transfusions and fasciotomy for compartment syndrome as well as a shortened length of stay in PICU. The onset of acute kidney injury was observed in 18 cases but restored in most patients within 48 h (77%). Five children died of which only two had received delayed antivenom immunotherapy due to its unavailability and deferred hospital admission.

**Conclusion:**

The advent of specific serotherapy has made it possible to optimize the management of patients and to prevent and treat local and systemic complications thus improving the overall prognosis; nevertheless, primary prevention remains the key to reducing snakebites morbimortality.

## Highlights

• Snakebites are a leading cause of mortality and permanent disabilities especially among children.
• Morocco represents a hot spot in northern Africa for venomous snakes where there is a wide variety of species.
• The clinical presentation is widely variable and may rapidly evolve in time.
• Snakebites are eminently preventable and manageable through community awareness and unified therapeutic action.
• The inaccessibility to healthcare structures and the occasional availability of immunotherapy continue to be the main limitation.

## 1 Introduction

Snakebite envenoming has been declared as a neglected tropical disease since 2017 by the World Health Organization and is still counted as a global health problem. It represents a leading cause of mortality and permanent disabilities especially among children in rural undeveloped areas. Worldwide statistics reach up to 400 000 victims with disabilities per year and more than 138 000 annual deaths [1].

Due to its climate and geographical entity, Morocco is prone to a variety of reptile species that differ from the northern and southern regions. The local ophidian fauna is mainly made of five species, two of which are venomous: the Elapidae, represented by the Naja Legionis which causes a neurotoxic syndrome leading to respiratory paralysis, and the Viperidae responsible for a viperine syndrome associating necrosis and hemorrhagic syndrome that can endanger vital and functional prognosis.

Despite insufficient epidemiological data and under-notification, national studies show the extent of snakebite cases and complications over the last decade. The annual incidence is 0.2 per 100 000 inhabitants with 30% of the cases involving children less than 15 years of age and a global mortality rate of 4% [2]. These statistics have encouraged a national prevention program with mandatory case notification and unified therapeutic management. Efforts have also been made to improve access to specific serotherapy but remain insufficient [3].

Our aim is to describe and compare the aspects of pediatric snakebite envenoming in a tertiary university hospital of the southern region of Morocco with a high envenomation incidence before and after the advent of antivenom serotherapy.

### 1.1 Patients and methods

We conducted a retrospective descriptive comparative study of children admitted to the pediatric intensive care unit in the university hospital center of Marrakech-Morocco following a snakebite envenomation over a period of eleven years from January 2010 to December 2020. The study period included two phases: before and after the accessibility to antivenom therapy in 2013.

Inclusion criteria were children under fifteen years old, as of restrictive hospital admission policies, admitted to the emergency department with local or systemic signs of snakebite envenoming as described in Table 1. We excluded patients with unavailable data or admitted with a dry bite (no envenoming) corresponding to the absence of local and general symptoms 24 h after a snakebite.

**Table 1:** Case definition of patients with snakebite envenoming [4].

No envenomation	Fang marks
	No local edema, no bleeding, mild pain
Mild envenomation	Severe pain, local edema (around the site of bite),no bleeding
	No systemic signs
Moderate envenomation	Regional edema (involving the major part of the limb)
	Blisters, localized necrosis
	and/or moderate systemic symptoms (tachycardia, nausea, diarrhea)
Severe envenomation	Extensive edema (spreading to the trunk) or necrosis
	Severe systemic symptoms (hypotension, shock, bleeding)

All patients responded to protocolized primary care based on the initial pediatric assessment triangle (appearance, breathing, and circulation) followed by early intravenous access and fluid therapy. Patients were systematically admitted to the pediatric intensive care unit following a venomous snakebite.

The distribution of serotherapy has been available in Morocco since 2013 with the acquisition of three antivenoms: FAV-Afrique®, Favirept®, and Inoserp-Mena®. In our series, only FAV-Africa® was available from 2013 to 2015, while Inoserp-Mena® was used since 2016. It was administered, when available, to all children less than 25 Kilograms, if signs of moderate or severe envenomation or in a bite to the head or neck regardless of body weight according to the national protocol [3].

Data was collected through medical charts and included demographic aspects, clinical presentation, therapeutic management, and outcomes. Statistical data analysis was performed using IBM SPSS Statistics, software version 20.0 X (IBM Corp., Armonk, NY, USA). Univariate and bivariate analyses were performed to assess the impact of the national treatment protocol and antivenom therapy in the reduction of functional morbidity and mortality, a p-value of less than 0.05 was considered statistically significant.

## 2 Results

This case series has been reported in line with the PROCESS criteria [5].

A total of seventy-five cases were enrolled in the study after retrieving two cases for not meeting inclusion criteria and one for missing data.

### 2.1 Demographics

Patients ranged in age from 8 months to 14 years old with a mean age of 10 years. Fifty-two were males (70%).

Admissions mainly included patients from rural areas of the southern region of Morocco after being attended and transferred from a regional health center. The average time to admission for our patients ranged from one to 72 h, whereas delayed admission time (later than 12 h after bite) involved 48% of cases. This was often due to difficult geographical access and the lack of adequate medical transportation methods.

Most cases occurred during daylight in the summer and spring seasons with a frequency of 65% and 24% respectively. Children were mainly playing outdoor activities with no protective shoes and had accidental (85%) or intentional (15%) contact with the snake. Bites more often took place distally in the lower limbs (80%). No cases of bites in the head or neck were observed. The snake type was identified by twelve children with the help of local iconography according to the incident location. All snakes belonged to the Viperidae family: four Echis leucogaster, six Vipera latastei and two Cerastes cerastes, In our series, ten children reported similar previous cases of envenomation in siblings and four of these accidents occurred in the same year. 44% of the patients underwent at scene ancestral practices consisting of incisions, scarifications, suction, or local application of plants or chemicals to the skin.

### 2.2 Clinical assessment

**Fig. 1:**
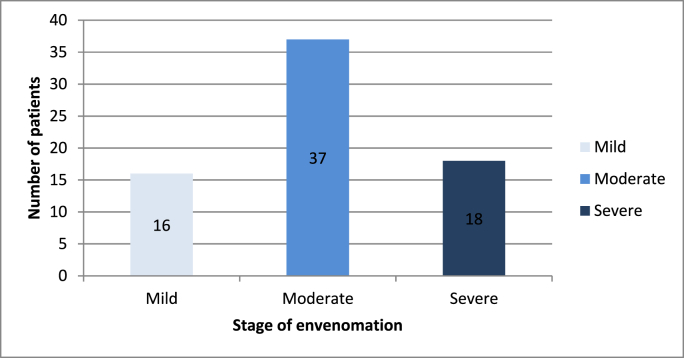
Classification of cases according to stage of envenomation.

**Fig. 2:**
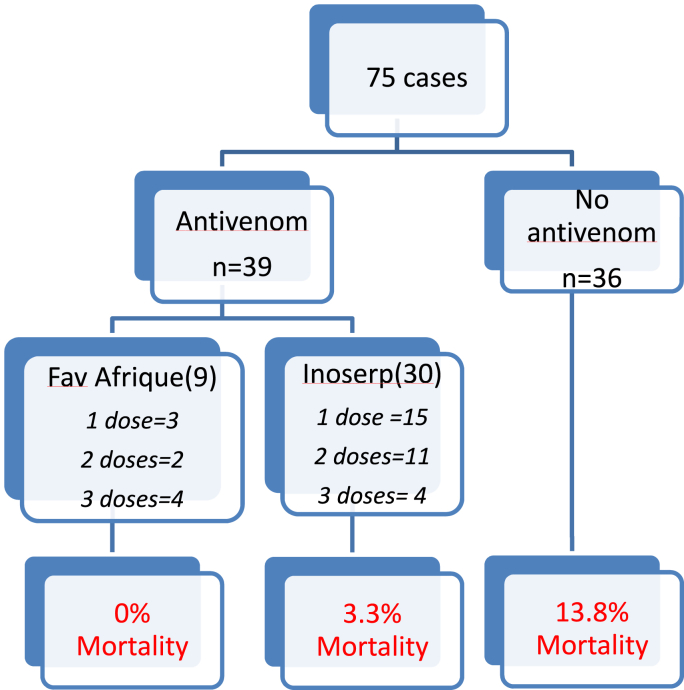
Lower limb non extensive edema with bruising and blisters due to a snakebite.

The majority of cases were classified as moderate (Fig. 1). Sixty-three children (84%) reported pain, bruising, and edema as the main local symptoms at admission (Fig. 2), whereas systemic manifestations included tachycardia (81.3%), hypotension (20%), vomiting, and/or diarrhea (34.6%). Hemorrhagic manifestations affected 32 patients consisting of epistaxis (n = 16), gingival bleeding (n = 12), and upper gastrointestinal bleeding (n = 4).

### 2.3 Biological results

The first hemostasis disorder to be seen is rapid onset of thrombocytopenia present at admission followed by a decrease by the first 24 h in prothrombin levels <50% involving 30 patients and a prolonged APTT in 24 patients that resolve after day 2; whereas dosages of fibrinogen remained within normal values.

Thrombocytopenia was observed in fifty patients (66.6%) and severe thrombocytopenia defined by a platelet count lower than <50 000 elts/mm3 was noted in twenty cases (26.6%). It is the first abnormality to resolve after antivenom administration but has also shown tendencies to subsequently decrease again. This should incite continuous monitoring of platelets even if normalized after antivenom administration for at least 48 h afterward.

Blood count also showed the onset of moderate anemia by the first 24 h in forty cases (52.2%) and a pronounced PNN- predominant leukocytosis upon admission in forty-five patients (58%) that tend to normalize before day 2.

Other frequent abnormalities included delayed rhabdomyolysis after the first 48 h with a peak by day 5 (55%), metabolic acidosis at admission (55%), and acute kidney injury (24%). Elevated liver enzymes were noticed in only five cases (Table 2).

**Table 2:** Biological results and their kinetics through hospital stay in patients with and without antivenom therapy.

		All cases N = 75	With Antivenom N = 39	Without Antivenom N = 36	p value
Prothrombin time (%)	Admission	71.2 ± 17.2	71.4 ± 16.9	70.5 ± 21.4	
	Day 1	69.4 ± 20.8	80.5 ± 19.7	69 ± 21	0.109
Cephaline Kaoline Time	Admission	31.7 ± 12.8	30.4 ± 13.6	38.4 ± 9.1	
	Day 1	31.6 ± 13.1	29.7 ± 8.6	31 ± 5.1	0.807
White cell count	Admission	18455 ± 8460	17748 ± 7951	21995 ± 9339	
	First 48 h	12311 ± 7161	11202 ± 7227	18556 ± 1131	0.028
Neutrophil count (/mm^3)^	Admission	15040 ± 7329	14725 ± 6924	18812 ± 8590	
	First 48 h	9590 ± 6590	9580 ± 4610	16690 ± 5449	0.004
Lymphocyte count (/mm^3)^	Admission	2352 ± 2443	2449 ± 2683	1890 ± 470	
	First 48 h	2425 ± 888	3103 ± 3541	2263 ± 1110	0.54
Platelet count (10^3^/mm^3)^	Admission	167 ± 129	180 ± 130	104 ± 111	
	Day 1	209 ± 119	237 ± 101	70 ± 14	0.003
	Day 2	229 ± 93	243 ± 91	101 ± 26	0.118
Hemoglobin (g/l)	Admission	12.8 ± 1.9	13.07 ± 1.88	11.9 ± 2	
	Day 1	11 ± 2.7	11.43 ± 2.47	8.5 ± 3.8	0.089
	Day 2	10.4 ± 2.2	10.9 ± 2.07	8.1 ± 2	0.044
Sodium (mmol/l)	Admission	136.2 ± 7.2	136.6 ± 6.3	134.5 ± 9.8	
	Day 1	135 ± 6.8	135.7 ± 7.1	134 ± 5.1	0.691
Potassium (mmol/l)	Admission	4.04 ± 0.58	4.11 ± 0.62	4 ± 0.3	
	Day 1	4.4 ± 0.5	4.51 ± 0.65	4.1 ± 0.6	0.515
Calcium (mg/l)	Admission	85.7 ± 9.8	86.4 ± 9.9	81.3 ± 9.4	
	Day 1	85.1 ± 6.9	85.9 ± 8.4	83 ± 1.4	0.639
Bicarbonates (mmol/l)	Admission	17.55 ± 4.07	18.05 ± 4.11	14.3 ± 1.52	
	Day 1	21.4 ± 2.77	21.6 ± 2.93	19.9 ± 0.9	0.071
Glucose (g/l)	Admission	1.88 ± 0.67	1.86 ± 0.63	1.9 ± 0.75	
	Day 1	1.53 ± 0.6	1.43 ± 0.53	1.61 ± 0.45	0.630
Blood urea (g/l)	Admission	0.3 ± 0.2	0.3 ± 0.11	0.4 ± 0.13	
	First 48 h	0.32 ± 0.15	0.34 ± 0.19	0.38 ± 0.11	0.584
Creatinine (mg/l)	Admission	3.6 ± 1.2	3.58 ± 1.34	4.15 ± 0.85	
	First 48 h	3.9 ± 2.4	3.83 ± 2.56	4.3 ± 2.36	0.772
Alanine Aminotransferase (U/L)	Admission	15.4 ± 6	14.1 ± 6.3	18 ± 1.4	
	First 48 h	26.4 ± 33	24.3 ± 31	48 ± 43.8	0.095
Aspartate Aminotransferase (U/l)	Admission	22.4 ± 8	25.1 ± 8.3	17.5 ± 10.6	
	First 48 h	117 ± 330	113 ± 346	152 ± 169	0.813
Fibrinogen (g/l)	Admission	2.7 ± 0.5	2.6 ± 0.5	No data	–
Creatin Kinase (U/l)	Admission	225 ± 137	227 ± 144	217 ± 106	
	Peak	2858 ± 5740	896 ± 1588	3476 ± 5086	0.039
Troponin (ng/l)	Admission	2.6 ± 1.03	2.6 ± 1.03	No data	–
C Reactive Protein(mg/l)	Admission	19.8 ± 61	19.3 ± 62.7	30 ± 5	
	First 48 h	26.6 ± 25	34.8 ± 58	70 ± 21	0.805

### 2.4 Complications

Local complications associated with Viperidae envenomation are predominated by compartment syndrome and were observed in 31 cases (41.3%). The diagnosis was based on clinical manifestations such as painful extensive edema, paresthesia, paralysis, pallor, and pulselessness. Only 5 patients had delayed compartment syndrome occurring 36 h after the bite. Limb diameter, pulse oximeter, and non-invasive infrared spectroscopy (NIRS) were used to monitor the extension as there was no available invasive monitoring of compartment pressure.

Systemic complications included hemodynamic instability (10%), respiratory distress (8%), and impaired consciousness (5.6%). The main organ failure was represented by acute kidney injury (AKI) in 18 cases, defined according to the Kidney Disease Improving Global Outcomes classification (KDIGO). AKI was associated with arterial hypotension (45%), oliguria (81%), coagulopathy (90%), and high white blood cell counts and neutrophil levels (81%). After effective fluid therapy and adequate primary resuscitation, renal function was restored in 14 patients, and only one patient required intermittent hemodialysis. The mortality rate among children with AKI was significantly higher than those who did not develop AKI (17% vs. 3% p = 0.04)

### 2.5 Non-specific treatment

The therapeutic management mainly consisted of symptomatic treatment because of the seasonal unavailability of the specific immunotherapy. After rapid initial assessment and conditioning, the use vasopressors (Norepinephrine) was necessary for nine patients due to hemodynamic instability despite adequate fluid resuscitation whereas mechanical ventilation was required for eight patients with respiratory distress.

Red blood cell transfusion was considered necessary in nineteen cases, fresh frozen plasma in ten cases, and platelet concentrates were administered to nine patients.

Effective multimodal analgesia was a mainstay of the management of these patients; the molecules used in our series of patients were paracetamol and morphine. Non-steroidal anti-inflammatory drugs have been ruled out as well as for corticosteroids.

Antibiotic therapy with amoxicillin-clavulanic acid was prescribed in 88% of cases either as prophylaxis or systematically given after a surgical fasciotomy. Tetanus prevention was carried out in all patients with subcutaneous tetanus serotherapy and local wound care.

### 2.6 Specific treatment: immunotherapy

**Fig. 3:**
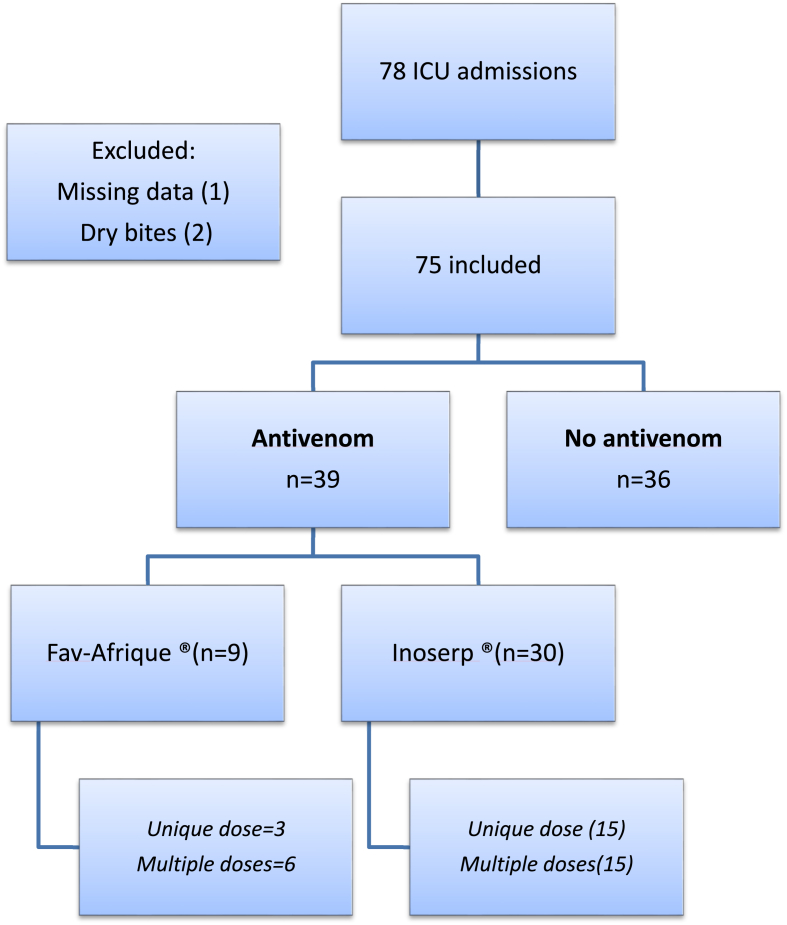
Flow diagram of patients admitted to the pediatric intensive care unit following a snakebite.

Thirty-nine patients were able to benefit from specific immunotherapy treatment, given the occasional availability of the product throughout the study period. Antivenoms were administered intravenously with no recorded adverse effect (Fig. 3). Additional doses were determined by clinical evolution and biological disorders after 6 h of the first administration and afterward.

Data regarding the time of antivenom administration was not available in 20 patients; hence, the mean bite to needle time in the remaining cases was 6 h ± 2.

### 2.7 Adjuvant surgical approach

Appropriate and non-delayed surgical intervention was performed in 37% of cases. Fasciotomy was needed in twenty-five patients with compartment syndrome, while limb amputation was performed in four patients (one mid-leg, one at wrist level and two at the toes) and cutaneous necrosectomy was required in eight cases.

The overall rate of limb amputation and fasciotomy without antivenom therapy was 11.4% and 44% respectively, reduced to 3.5% and 30.7% after antivenom therapy use (Table 3).

**Table 3:** Therapeutic management and outcome of patients before and after access to antivenom therapy.

	No antivenom n = 36	Antivenom therapy n = 39	p-value
Fasciotomy n (%)	17 (47%)	8 (20%)	0.014
Limb amputation n (%)	4 (11%)	1 (2.5%)	0.188
AKI n (%)	12 (33%)	6 (15%)	0.066
Red blood cells transfusion n (%)	13 (36%)	6 (15%)	0.039
Platelets transfusion n (%)	7 (19%)	2 (5%)	0.078
Mechanical ventilation n (%)	6 (16%)	2 (5%)	0.115
Mean duration of mechanical ventilation (days)	3.5	2.5	0.506
Patients with vasoactives drugs n(%)	7 (16%)	2 (5%)	0.062
Mean duration of vasoactive drugs (days)	2.7	2.5	0.808
Mean length of stay ICU (days)	3.73 ± 2.11	2.45 ± 1.45	0.007
Mortality	4 (13.8%)	2 (5%)	0.331

### 2.8 Outcome –evolution

The mean duration of stay was 3.5 days [1–8], children were either transferred to a pediatric surgical unit for follow-up or discharged. The overall mortality rate was 8%, following an Acute Respiratory Distress Syndrome (ARDS) or a multi-organ failure with refractory hemodynamic instability. Of the children who died, only two had received delayed antivenom immunotherapy (26 and 32 h after bite) due to its unavailability and deferred hospital admission.

Eight patients had long-term sequelae such as loss of cutaneous substance that ultimately required a skin graft and one patient presented with persistent reduced mobility with clawed fingers.

## 3 Discussion

Snakebite envenoming is a potentially life-threatening disease frequently encountered in rural areas of developing countries. Their severity lies in the complexity of snake venoms that contain more than 100 different toxic and non-toxic proteins and peptides that have specificity for a wide range of tissue receptors, making them clinically challenging with broad features and complications [6].

Although snake bites are seen at all ages, the pediatric population is at a higher risk and accounts for a significant proportion of envenomation cases with a rate of 30%, with a correlation between age group and fatal evolution: children aged 5–9 years are 3.37 times more at risk than other age categories in Morocco [7].

As in most studies, male dominance is asserted. The bite has a seasonal distribution as they classically occur in the daytime during the summer season and more rarely in the evening. At-risk behaviors include outdoor activities in the fields and bushes, and the bites are more frequently located at limb extremities [8]. In our study, the lower limbs were most affected by their proximity to the ground and therefore the snake, but all parts of the body may be concerned [9].

Morocco represents a hot spot in northern Africa for venomous snakes where there is a wide variety of species with seven types belonging to the viper family (Daboia mauritanica, Bitis arietans, Cerastes cerastes, Cerastes Vipera, Echis leucogaster, Vipera latastei, Vipera monticola), and one to elapids (Naja haje legionis) [10]. These species are venomous and deadly dangerous for man; their identification has been facilitated by the use of iconography and descriptive specific features in all medical institutes across the country [7].

The location of this study took place in the region of Marrakech-Tensift-Elhaouz where the prevalent viper species is Daboia mauritanica, also known as Vipera lebetina mauritanica; along with *C. cerastes*, they represent one of the most virulent species [11].

The clinical presentation is widely variable and may rapidly evolve in time. Symptoms depend on the involved species and the severity of envenomation [12]. We often distinguish the Cobraic syndrome responsible for neurotoxicity and the Viperic syndrome, more frequent, which is responsible for capillary leak syndrome and coagulation disorders. At clinical examination, painful edema is usually constant and can progress to cause compartment syndrome [13]. General symptoms often include diarrhea and vomiting that can add up to the hypovolaemia state. Arterial hypotension can be observed due to major hypovolaemia by severe vasodilatation and capillary leakage along with direct myocardial cardiotoxicity leading to a cardiovascular collapse. In addition, biological abnormalities add up to the clinical severity with low prothrombin levels and platelets as detected in our patients. All of these clinico-biological manifestations find their explanation in the pharmacotoxic properties of snake venom which is a complex of protein components with enzymatic activity that can induce various direct pharmacological effects: hemorrhagic, hemolytic, cardiotoxic, neurotoxic, procoagulant, anticoagulant, and ion channel inhibitors [14,15].

The commonly used clinical classification based on symptoms and clinical examination corresponds to Audebert et al.'s [16]. Severity depends on the volume of the injected venom and its extent of absorption; this explains why most studies demonstrate a higher severity of envenomation in children when compared to adults since they have a lower body surface and thus a greater venom concentration [14,17].

While many victims’ relatives still resort to ancestral harmful methods such as scarifications suction and local topics application, these widespread practices must be proscribed and prompt access to care should be encouraged.

The treatment of moderate and severe viperin envenomation is mainly based on antivenom immunotherapy, the indications of which are currently well codified and combined with strict clinical-biological monitoring without delaying a surgical procedure if necessary such as a fasciotomy or necrosectomy [18].

FAV-Afrique® was the first antivenom therapy available in Morocco since 2012, which is mainly directed against Bitis arietans, Echis, and Naje haje. It was later replaced by Inoserp®-PanAfrica, an F(ab’)2 polyvalent antivenom with high immunotherapeutic specificity and a wider spectrum of action indicated in the case of envenomation by Naja nigricollis, Dendroaspis polylepis, Echis ocellatus and Bitis arietans, in addition to Viperids (Echis leucogaster, Echis pyramidum, Bitis gabonica rhinoceros, Bitis gabonica) and Elapidae (Dendroaspis viridis, Dendroaspis angusticeps, Dendroaspis jamesoni, Naja melanoleuca, Naja haje, Naja pallida, Naja nivea, Naja katiensis). It has shown efficacy and tolerance in various epidemiological settings [19]. The national protocol recommends the intravenous administration of one to two vials of antivenom diluted in 5–10 mL/kg of 5% dextrose solution over 1 h and warns of the rare but probable risk of anaphylactic shock.

Our study has demonstrated the benefice of antivenom therapy in decreasing the need for blood transfusion, fasciotomy, and duration of ICU stay. Mortality and systemic complications were lower with specific immunotherapy but were not statistically significant in this small patient sample. Other more exhaustive non pediatric studies have been able to display the importance of antivenom therapy in reducing mortality and disabilities [20].

Replacement treatments (platelet transfusion, fibrinogen, and fresh frozen plasma) are not effective because they are consumed by the procoagulant venom toxins that remain active for several days in the victim's bloodstream. However, blood transfusion could be an awaiting solution while obtaining the specific therapy serum. Thus, the WHO recommends the transfusion of labile blood products whenever serious coagulation disorders are present with uncontrolled bleeding in the absence of specific anti-venom [21].

The short-term outcome is predominated by the rapidity and adequacy of symptomatic and specific management whereas long-term sequelae with lost or altered functions of limbs remain the major burden for these victims [22]. Therefore, the WHO has recently launched a prevention strategy and control program in 2019 in order to reduce by 50% the global mortality and morbidity rate by 2030 with the “Make antivenom accessible to all” campaign [1,23].

## 4 Conclusion

Endowed with significant morbidity and mortality, Ophidian envenomation remains a public health challenge. Its prognosis has drastically changed owing to antivenom serotherapy, the only specific treatment that has confirmed its benefit in the rapid restoration of hemostasis disorders and the reduction of local complications.

Snakebites are eminently preventable and manageable through community awareness and unified therapeutic action, consistent with the degree of medical and surgical urgency. Evolution is favorable in most cases; however, the inaccessibility to healthcare structures and the occasional availability of immunotherapy continue to be the main limitation.

## Ethical approval

Written informed consent was obtained from the patient for publication of this case report and accompanying images. A copy of the written consent is available for review by the Editor-in-Chief of this journal on request.

## Sources of funding

The authors declared that this study has received no financial support.

## Author contribution

FAJRI MOHAMED: Corresponding author writing the paper. Youssef mouaffak: Correction of the paper.

## Registration of research studies

1. Name of the registry:
2. Unique Identifying number or registration ID:
3. Hyperlink to your specific registration (must be publicly accessible and will be checked):

## Guarantor

Fajri mohamed.

## Consent

Written informed consent was obtained from the patient for publication of this case report and accompanying images. A copy of the written consent is available for review by the Editor-in-Chief of this journal on request.

## Provenance and peer review

Not commissioned, externally peer reviewed.

## Declaration of competing interest

Authors of this article have no conflict or competing interests. All of the authors approved the final version of the manuscript.
